# Functional analysis and development of a CRISPR/Cas9 allelic series for a CPR5 ortholog necessary for proper growth of soybean trichomes

**DOI:** 10.1038/s41598-019-51240-7

**Published:** 2019-10-14

**Authors:** Benjamin W. Campbell, Jacob W. Hoyle, Bruna Bucciarelli, Adrian O. Stec, Deborah A. Samac, Wayne A. Parrott, Robert M. Stupar

**Affiliations:** 1 Department of Agronomy and Plant Genetics, University of Minnesota, St. Paul, MN USA; 2 Department of Crop and Soil Sciences, University of Georgia, Athens, GA USA; 3 USDA-ARS-Plant Science Research Unit, St. Paul, MN USA; 4 Department of Plant Pathology, University of Minnesota, St. Paul, MN USA

**Keywords:** Plant genetics, Plant molecular biology

## Abstract

Developments in genomic and genome editing technologies have facilitated the mapping, cloning, and validation of genetic variants underlying trait variation. This study combined bulked-segregant analysis, array comparative genomic hybridization, and CRISPR/Cas9 methodologies to identify a *CPR5* ortholog essential for proper trichome growth in soybean (*Glycine max*). A fast neutron mutant line exhibited short trichomes with smaller trichome nuclei compared to its parent line. A fast neutron-induced deletion was identified within an interval on chromosome 6 that co-segregated with the trichome phenotype. The deletion encompassed six gene models including an ortholog of *Arabidopsis thaliana CPR5*. CRISPR/Cas9 was used to mutate the *CPR5* ortholog, resulting in five plants harboring a total of four different putative knockout alleles and two in-frame alleles. Phenotypic analysis of the mutants validated the candidate gene, and included intermediate phenotypes that co-segregated with the in-frame alleles. These findings demonstrate that the *CPR5* ortholog is essential for proper growth and development of soybean trichomes, similar to observations in *A*. *thaliana*. Furthermore, this work demonstrates the value of using CRISPR/Cas9 to generate an allelic series and intermediate phenotypes for functional analysis of candidate genes and/or the development of novel traits.

## Introduction

Irradiation mutagenesis can be easily and effectively used to generate large numbers of unique mutants, but the resulting individuals can be recalcitrant to candidate gene identification due to the types of mutations generated. Genomic investigations of irradiation-generated mutants in soybean and other species have recently been conducted using array comparative genomic hybridization (aCGH) and whole genome sequencing. Investigations of soybean fast neutron (FN) mutant lines have found evidence of various genomic structural variants in these lines, including deletions, duplications, inversions, and translocations^1–10^. While most phenotypes observed in FN plants appear to be caused by large deletions encompassing multiple genes, it is not possible to recover genetic recombinants within these deletions, making it difficult to ascertain the causative gene underlying a phenotype. A recent study examining a soybean FN mutant with elevated steric acid levels demonstrated that the list of candidate genes in a deleted interval could be reduced by comparing overlapping mutations from multiple mutant individuals that shared the elevated steric acid phenotype^7^. However, this approach requires multiple genotyped and phenotyped mutant individuals. Even with many genotyped and phenotyped mutant individuals, it can still be difficult to find the necessary set of individuals with overlapping and or non-overlapping deletions to definitively identify the candidate gene.

Early work on the mapping and characterization of FN mutants in soybean has typically focused on traits exhibiting qualitative segregation patterns and oftentimes observable morphological phenotypes. One recent example focused on identifying a gene that causes a gnarled trichome phenotype^4^. Trichome mutants have been well characterized in model plant systems, with several studies investigating morphology and cellular structures in *Arabidopsis thaliana*. Leaf trichome cells often undergo several rounds of endoreduplication, in which the nuclear genome replicates in the absence of mitosis, leading to elevated nuclear gene content. Several genes in *A*. *thaliana* have been reported to affect trichome morphology, and show accompanying deviations from normal trichome ploidy levels: *GLABRA1* (*GL1*)^11,12^, *TRANSPARENT TESTA GLABRA1* (*TTG*)^13^, *TRIPTYCHON* (*TRY*) and *GL3*^12,14^; *RASTAFARI* (*RFI*) and *POLYCHOME* (*PYM*)^15^; cyclin-dependent kinase inhibitor proteins (ICK/KRPs)^16^; *SPINDLY* (*SPY*)^15,17^; *ROOT HAIRLESS2* (*RHL2*) and *HYPOCOTYL6* (*HYP6*)^18,19^; *CONSTITUTIVE EXPRESSION OF PR GENES 5* (*CPR5*) also known as *HYPERSENESCENCE* *1* (*HYS1*)^20–23^; *KAKTUS* (*KAK*)^12,24,25^; and *SIAMESE* (*SIM*)^26,27^. In summary, a large body of literature in *A*. *thaliana* has established a relationship between altered trichome morphology and perturbations to normal trichome endoreduplication levels.

In this study, we were interested in mapping and cloning the gene responsible for a short trichome phenotype in the soybean FN mutant R59C46. Bulked-segregant analysis (BSA) and aCGH quickly identified an ortholog of *CPR5* as a candidate gene. We were furthermore interested in testing the use of CRISPR/Cas9 as a validation tool to overcome the difficulties of identifying and validating causative genes located in deleted intervals. CRISPR/Cas9 was successfully used to generate an allelic series of *CPR5* knockout and in-frame mutations. Characterization of these materials validated that the soybean *CPR5* ortholog is necessary for proper soybean leaf trichome development.

## Results

### The R59C46 trichomes are shorter and have decreased nuclear size compared to wild-type

**Figure 1:**
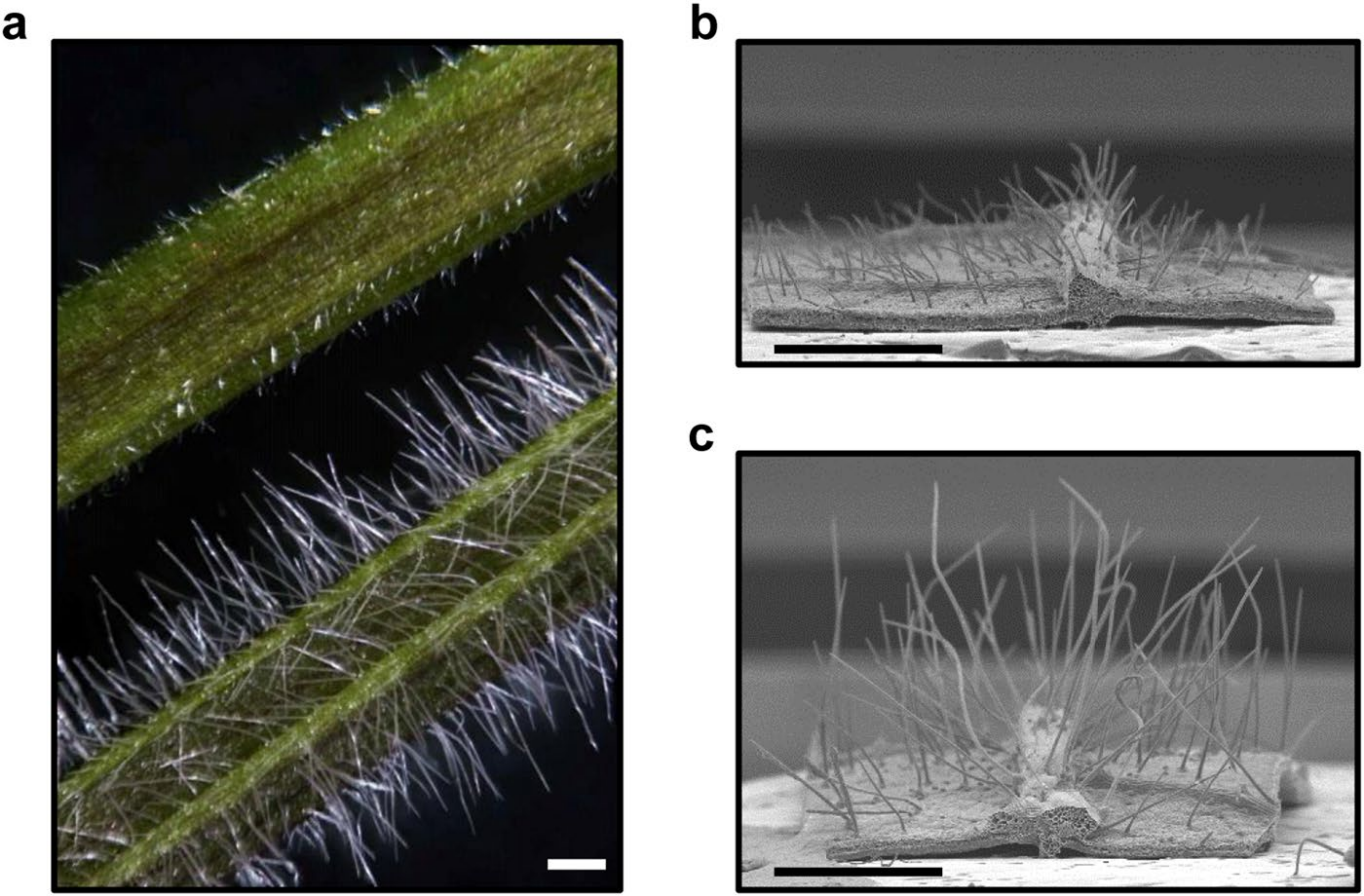
Phenotypes of the short trichome mutant (R59C46) and wild-type parent (M92-220) soybeans. (**a**) Trichome phenotypes of the mutant (top) and wild-type (bottom) on leaf petioles. SEM images of (**b**) mutant and (**c**) wild-type leaf trichomes show differences in trichome morphology. The size bar in each image is 1 mm.

**Figure 2:**
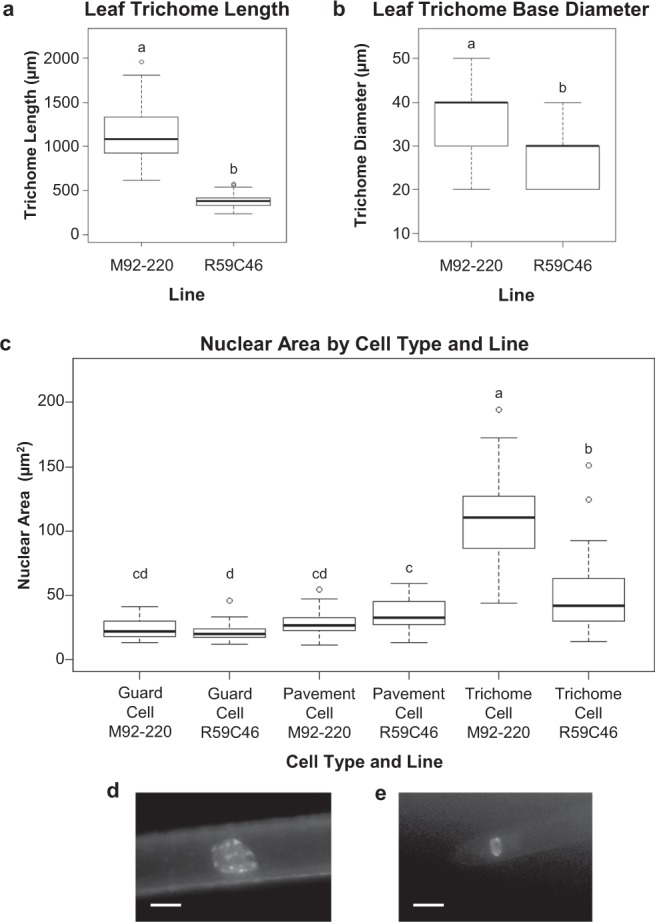
Nuclear area size differences between wild-type (M92-220) and mutant (R59C46) leaf trichome cells underlie differences in leaf trichome length. Differences between the mutant and wild-type were also observed for the cell size of the leaf trichomes, as measured by lengths (p < 0.001) (**a**) and base diameter (p < 0.001) (**b**). (**c**) Nuclear size differences were not significant between the leaf guard cells (p = 0.989) or between the leaf pavement cells (p = 0.504), respectively, for M92-220 or R59C46. However significant size differences were observed between the leaf trichome cell nuclear area sizes of M92-220 and R59C46 (p < 0.001). Representative images of M92-220 (**d**) and R59C46 (**e**) trichome cell nuclei, indicating the size difference between the genotypes. The size bar in both images is 10 µm.

The FN mutant R59C46 exhibiting short trichomes (SOY:0001804) and thin trichomes (SOY:0002236) was identified in the Soybean Fast Neutron Mutant Population developed at the University of Minnesota^1,2^ (Figs 1 and 2a,b). The leaf trichomes of the mutant were shorter (p < 0.001) and thinner (p < 0.001) than those of the FN parent M92-220. Preliminary assessments of leaf trichome density and the size and shape of epidermal pavement cells did not show any obvious differences between the R59C46 and M92-220 plants (data not shown). Furthermore, roots hairs of the R59C46 and M92-220 plants were examined, but no differences in root hair length were observed (data not shown). However, the R59C46 mutant exhibited reduced above-ground growth and stature compared to M92-220 in greenhouse conditions, and these mutant phenotypes were strongly observed in field conditions (see Supplementary Fig. S1).

The nuclear areas of the mutant and the wild-type lines were compared to investigate if the shortened R59C46 trichomes also exhibited a reduction in the size of the nucleus within the trichomes (GO:0042023). Measuring the relative fluorescence of DAPI-stained nuclei has been previously used as a method of assessing nuclear DNA content^22,25^. An analysis of leaf cell nuclear area found that there were no differences between R59C46 and M92-220 for both guard cells (p = 0.989) and pavement cells (p = 0.504) (Fig. 2c). However, the nuclear areas of R59C46 and M92-220 leaf trichomes were significantly different (p < 0.001), with the nuclear area of M92-220 being on average twice the size of the R59C46 nuclear area (Fig. 2c–e). These results demonstrate that on average R59C46 trichome cell nuclei are smaller and might experience fewer rounds of endoreduplication than the wild-type M92-220 trichome cell nuclei.

### The trichome phenotype maps to a FN-induced chromosome 6 deletion encompassing six gene models

A cross between R59C46 and genotype ‘Noir 1’ resulted in an F_1_ individual that displayed wild-type/normal trichomes (data not shown). The subsequent F_2_ progeny segregated 68:28 (wild-type: mutant), following a 3:1 segregation ratio (chi-squared p-value = 0.346). These results indicated that the mutant phenotype was caused by a recessive mutation that segregated as a single locus.

**Figure 3:**
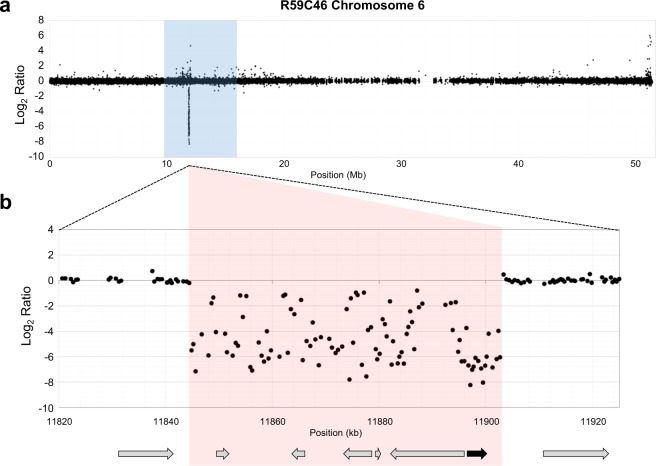
Identification of the causative deletion using aCGH. (**a**) Bulked segregant analysis was used to map the short trichome phenotype to a 7.28 Mb region on chromosome 6, which is shaded in blue. A single deletion was identified in the mapping interval using aCGH. The cluster of probes with log_2_ ratios below 0.0 indicate an absence of DNA in the mutant (R59C46) compared to the wild-type (M92-220). (**b**) Primers were designed to span the deletion and amplicons were sequenced to identify the exact breakpoint edges. The deleted interval is shaded in red. The deletion encompassed six gene models, indicated in the diagram by arrows. The candidate gene *GmCPR5* (Glyma.06g145800) is indicated by a solid black arrow.

Genetic mapping was conducted to locate the causative locus. BSA using the SoySNP6K Beadchip mapped the mutation to a 7.28 Mb interval on chromosome 6 (Gm06: 9,087,027-16,365,309) containing 755 genes in the soybean genome (version Glyma.Wm82.a2.v1.1^28^). Detailed aCGH analyses identified four chromosomes with putative deletions (chromosomes 3, 6, 8, and 12) in the R59C46 mutant (see Supplementary Table S1). One deletion encompassing six genes on chromosome 6 (see Supplementary Table S2) overlapped with the interval mapped by BSA (Fig. 3). Primers were designed to span the deletion (see Supplementary Table S3), and sequencing of the PCR fragments found that the deletion was 58.6 kb in length (Gm06: 11,844,488-11,903,108). This was a simple deletion (see Supplementary Figs S2 and S3) with no insertions, duplications, inversions, or translocations, which have been found in other soybean FN mutants from this population^1,2,4,5^. A co-segregation test was conducted with 96 segregating F_2_ individuals to validate the mapping results. The wild-type PCR fragment amplified only in F_2_ individuals with wild-type trichomes, and individuals with mutant trichomes only amplified the deletion-spanning fragment (see Supplementary Fig. S2). These segregation results are consistent with the hypothesis that the identified deletion contains the causative gene and requires the deletion to be homozygous to exhibit the mutant phenotype.

### Validation of a *CPR5* ortholog using CRISPR/Cas9

**Figure 4:**
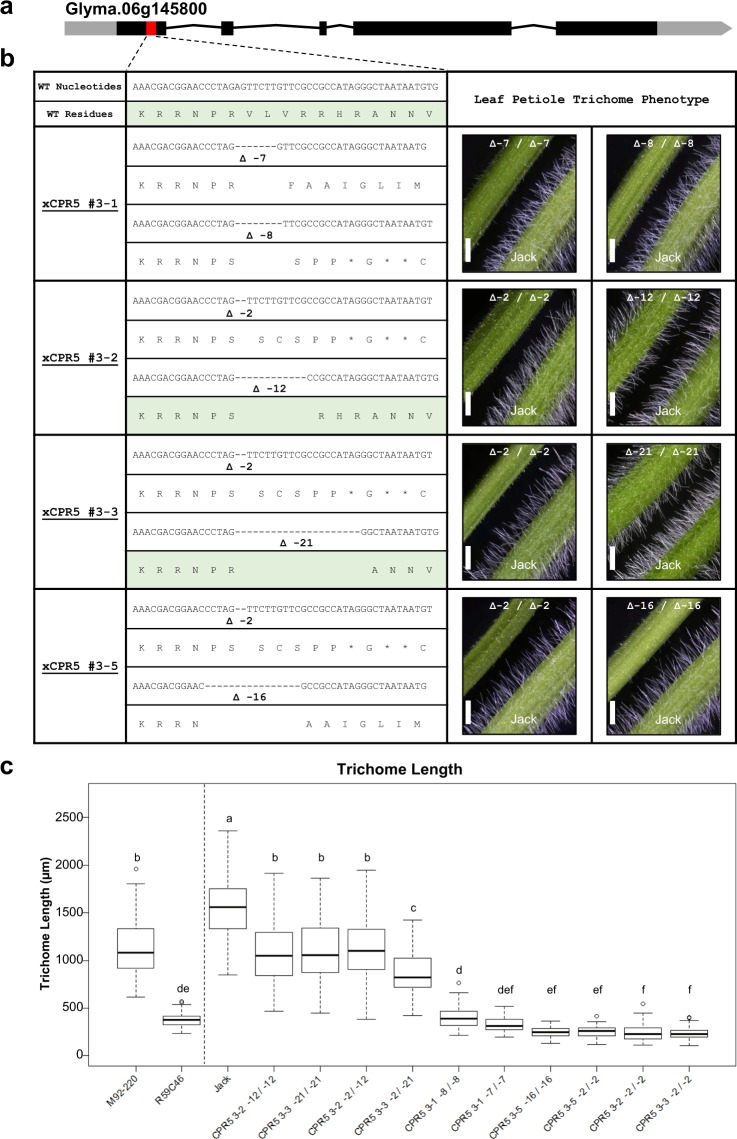
CRISPR/Cas9 mutation of *GmCPR5* (gene model Glyma.06g145800) and phenotypic validation of the candidate gene. (**a**) A gene model of *GmCPR5* with the CRISPR/Cas9 target site indicated with a red box. (**b**) Nucleotide and predicted translation sequences of *GmCPR5* at the CRISPR target site in a wild-type (WT) ‘Jack’ line and in four independently derived regenerated transgenic T_2_ families in the ‘Jack’ background, named xCPR5 #3-1, xCPR5 #3-2, xCPR5 #3-3, and xCPR5 #3-5. Each of the T_0_ plants, that gave rise to each T_2_ family, was found to have biallelic mutations when tested at 30 weeks after transformation. Some mutations were identical among the different plants, resulting in a total of six unique mutated alleles. Two alleles were in-frame deletions, indicated by green shading. All other alleles caused frameshifts. To the right of the sequences are leaf petiole trichome phenotypes of homozygous CRISPR-mutated T_2_ plants, compared to the ‘Jack’ control. Both alleles in the families xCPR5 #3-1 and xCPR5 #3-5 as well as the 2 bp deletion allele in families xCPR5 #3-2 and xCPR5 #3-3 were frameshift mutations and resulted in very short trichomes, indicating that complete disruption of *GmCPR5* results in shorter trichomes. In contrast, the in-frame deletions alleles of the families xCPR5 #3-2 (−12 bp) and xCPR5 #3-3 (−21 bp) resulted in phenotypes more similar to wild-type. The size bar in each image is 2 mm. (**c**) Trichome length measurements for the lines in the study: wild-type line M92-220, fast neutron mutant R59C46, wild-type line ‘Jack’, and ‘Jack’ derived CRISPR/Cas9 mutant lines. Lines to the left of the vertical dotted line are derived from M92-220 and lines to the right of the line are derived from the cultivar ‘Jack’. The frame-shift CRISPR/Cas9 mutant lines displayed trichome lengths similar to the fast neutron mutant R59C46 validating the gene’s function in trichome length. Uniquely, two homozygous in-frame deletions (−12/−12) and (−21/−21) displayed heritable intermediate trichome length phenotypes. These lines demonstrate that CRISR/Cas9 can be used to create in-frame deletions that result in intermediate phenotypes.

Among the genes within the deleted interval on chromosome 6 (see Supplementary Table S2), gene model Glyma.06g145800 was the strongest candidate gene, as loss of function mutants for the *A*. *thaliana* ortholog of this gene, known as *CPR5*, display altered trichome phenotypes^20–22^. This gene model (known as Glyma06g15080 in the Glyma.Wm82.a1.v1.1 genome assembly version) shows a relatively balanced transcription profile across a wide range of tissue types in wild type (genotype ‘Williams 82’) soybean^29^. To validate the candidate gene function, the soybean cultivar ‘Jack’ was transformed with a CRISPR/Cas9 T-DNA designed to generate mutations in the first exon of Glyma.06g145800. Five transgenic events were identified and Tracking of Indels by Decomposition (TIDE)^30^ analysis was used to determine the spectrum and frequency of mutations. Each event was confirmed to have some degree of editing, ranging from 48% to 99% of amplicons edited (see Supplementary Fig. S4). By week 10, event #3 showed evidence of being edited at 62.1%, and this event was chosen for plant regeneration, resulting in five plants. Two of the T_0_ plants (xCPR5 #3-1 and xCPR5 #3-2) were sampled again at 20 weeks (see Supplementary Fig. S5). Both xCPR5 #3-1 and xCPR5 #3-2 plants showed >90% biallelic editing, with all indels identified as being one of the two indels present in each plant (see Supplementary Fig. S5). All T_0_ plants were sampled again at 30 weeks, and all showed biallelic editing (Fig. 4). Four of the T_0_ plants (xCPR5 #3-2, #3-3, #3-4, and #3-5) shared the same two base pair deletion for one allele, while only two of these plants (xCPR5 #3-4 and #3-5) also shared the same second allele. As xCPR5 #3-4 and #3-5 shared the same bi-allelic mutations, only #3-5 is shown. Collectively, the five T_0_ plants produced from xCPR5 event #3 harbored four different allelic combinations, including six different edited alleles.

Genotyping the T_1_ progeny and T_2_ progeny confirmed that the alleles present in the T_0_ individuals were inherited to the T_2_ generation. Short trichome phenotypes were observed in the T_0_ and T_1_ plants, and detailed trichome length measurements were conducted in the T_2_ progeny in order to quantify the reduction in trichome length. Roughly three different trichome length phenotypic classes were observed in the mutant T_2_ families (Fig. 4b,c; see Supplementary Fig. S6). As the xCPR5 #3-4 and #3-5 families share the same bi-allelic mutations, only trichome length data were collected from the xCPR5 #3-5 family. The shortest trichomes were found on homozygous frame-shift alleles from all the T_2_ families, which displayed trichomes that were similar in length or shorter than those of the fast neutron mutant R59C46 (Fig. 4c). Plants harboring homozygous in-frame alleles (−12/−12 or −21/−21) or heterozygous in-frame/frame-shift allele combination (−2/−12 or −2/−21) exhibited intermediate trichome sizes, smaller than ‘Jack’ but larger than the plants with homozygous frame-shifts (Fig. 4c). The heterozygous combination with the longer in-frame deletion (−2/−21) resulted in trichome lengths that were slightly shorter than the homozygous in-frame or the heterozygous individuals with the other in-frame deletion (−2/−12). Regardless of allele type and combination, all CRISPR derived plants with bi-allelic mutations displayed shorter trichomes than wild-type ‘Jack’ indicating that disruptions to the *CPR5* ortholog reduce trichome length (Fig. 4b,c).

## Discussion

### The functions of CPR5 orthologs in soybean and *Arabidopsis thaliana*

This study combined classical genetic mapping, aCGH, and CRISPR/Cas9 induced mutations to identify Glyma.06g145800, an ortholog of *A*. *thaliana CPR5*, hereon referred to as *GmCPR5* (for *Glycine max CPR5*), as a gene necessary for proper trichome growth and development in soybean. While trichome morphology differs greatly between *A*. *thaliana* and *G*. *max*, the similarity in the overall reduction in trichome size for both *A*. *thaliana cpr5* mutants and the soybean fast neutron mutant R59C46 suggested that *GmCPR5* is the causative gene for the soybean short trichome phenotype. This hypothesis was validated using CRISPR/Cas9 targeted mutagenesis. The soybean reference genome contains only a single copy of *GmCPR5*, and thus disruption of the gene may be expected to result in an altered trichome phenotype.

The *A*. *thaliana* and soybean *cpr5* mutants have several features in common. In addition to the overall reduced leaf trichome size, both mutants display reduced nuclear sizes in the leaf trichome cells^22^. Furthermore, neither mutant shows clear disruptions to the number and spacing of trichomes across the leaf nor disruptions to root hair development^21^. However, some differences were also observed between the orthologous mutants. First, the leaf trichomes in the *A*. *thaliana* mutants were found to die and collapse, starting with 4-week old plants^22^, which was not observed in the soybean mutants. Furthermore, the *A*. *thaliana cpr5* mutant was reported to show a reduced nuclear size in epidermal pavement cells^22^, while our assessment of the *Gmcpr5* mutant did not display this phenotype.

The *A*. *thaliana CPR5* gene was first discovered for its role in disease resistance signaling pathways^20^. Specifically, *cpr5* mutants fall into a class of “disease lesion mimics” due to their propensity to respond as if under continual pathogen attack^20^. It has also been reported that *A*. *thaliana cpr5* trichomes have altered cell wall structure^23^, which may be related to its cellular morphology. It would be interesting to test the role of *GmCPR5* in soybean plant pathogen response. While *A*. *thaliana cpr5* mutants have been observed to have an altered plant-pathogen response^20^, the soybean mutants in this study were not purposefully challenged with pathogens. Anecdotally, we did not observe susceptibility to foliar disease for the R59C46 mutant in field, greenhouse, or growth chamber experiments. However, additional study is required to determine the role that *GmCPR5* plays in soybean plant-pathogen interactions.

### The utility of gene editing for functional validation and allelic diversification

This study described the creation of multiple induced mutations in *GmCPR5*. While *GmCPR5* seemed to be an excellent candidate gene in this study, further gene validation was necessary for two reasons. First, fast neutron irradiation has been shown to sometimes result in smaller mutations than can be detected by aCGH. Thus, it was possible that an undetected causative mutation could simply have been linked to the 58.6 kb candidate deletion on chromosome 6^31^. Second, genetic recombination is not possible in a deleted chromosome segment, and thus recombination cannot be used to narrow down the list of candidate genes within a deletion. While the 58.6 kb deletion associated with the mutant trichome phenotype only contains six genes, previous studies using irradiation mutants in soybean have found examples of lines carrying homozygous deletions encompassing over 100 genes and heterozygous deletions encompassing several hundreds of genes^1^. Thus, a method is needed for identifying and validating candidate genes from deleted segments. As a proof of concept for validating candidate genes in fast neutron mutants, CRISPR/Cas9 was used to induce mutations in the *GmCPR5* candidate gene in a wild-type line in order to generate mutants that resemble the mutant trichome phenotype of R59C46. Comparisons of the trichomes lengths of the T_2_ mutants and the wild-type line ‘Jack’ indicated that disrupting *GmCPR5* greatly reduced trichome length and thus mimicked the phenotype of R59C46 (Fig. 4b,c), validating the hypothesized candidate gene.

The CRISPR/Cas9 T-DNA was delivered by biolistic transformation and event #3 generated four unique biallelic mutant T_0_ individuals with six unique mutations in the candidate gene (four frame-shift deletions and two in-frame deletions). This finding suggests that our CRISPR/Cas9 reagent had a slow-but-steady activity while the cell lines were in tissue culture. The slow-but-steady activity can result in a single mutant allele being shared between multiple cell lineages if the mutation event occurred before subsequent lineage differentiation and prior to histodifferentiation in tissue culture (Fig. 4b). Thus, a pattern of shared alleles can be used to infer the order in which the mutations occurred. For instance, it is possible that the two base pair mutation that is shared between multiple T_0_ individuals occurred before the mutations to the second allele (Fig. 4b). The identical pair of alleles in xCPR5 #3-4 and #3-5 indicates that these specific editing events may have occurred prior to histodifferentiation in tissue culture.

The ability to obtain multiple alleles, including both frameshift mutations and in-frame mutations, is a clear advantage when assigning gene function. Intermediate phenotypes were observed in the homozygous in-frame deletions (−12/−12 and −21/−21) lines and the heterozygous combination (−2/−12 and −2/−21) lines (Fig. 4c). Alignment of CPR5 proteins from various species revealed that the in-frame deletions appear to have disrupted relatively conserved amino acids (see Supplementary Fig. S7). Deletion of these amino acids appears to have resulted in decreased, but not eliminated, *GmCPR5* function.

Most applications to date using CRISPR/Cas9 technology in crop plants has focused on creating gene knockouts, targeted gene insertions, and targeted gene edits (examples from soybean include^32–35^). Broadly speaking, these outcomes have focused on generating strong, qualitative and bi-modal phenotypes. One exceptional study was performed in tomato, where cis-regulatory regions were modified (rather than protein-encoding regions) to generate a quantitative spectrum of phenotypes for productivity traits^36^. In the present study, we have demonstrated the capacity to generate intermediate phenotypes by identifying targeted in-frame mutations within conserved protein-encoding sequences. Such mutations may generate novel phenotypes, as the loss of evolutionarily conserved amino acids may alter protein function, as was observed in this study. Site-Directed Nuclease technologies are unique as a mutagen in their ability to consistently generate indels that are large enough (>2 bp) to generate in-frame mutations without being so large as to eliminate gene function. Recent advances in CRISPR editing now provide better predictability of allelic outcomes based on the sequences targeted, which may enable the purposeful creation of in-frame mutations^37^. The ability to create intermediate phenotypes may be useful for studying genes that cause lethality in the homozygous knockout state. Furthermore, novel alleles with small in-frame deletions or modifications to regulatory elements may be useful for optimizing certain plant phenotypes and thus may have utility in crop improvement strategies, particularly for crop species with highly bottlenecked germplasm.

## Methods

### Populations and phenotyping

A soybean fast neutron mutant population was generated at the University of Minnesota^2^ using a soybean line ‘M92-220’ derived from the variety ‘MN1302’^38^. From this mutant population, the mutant R59C46 (Soybase mutant FN0175946) was identified as having trichomes that are shorter in length than wild-type trichomes (SOY:0001804) (Figs 1 and 2a) and thinner in width than wild-type trichomes (SOY:0002236) (Figs 1 and 2b). This mutant was included in previous characterizations of the population shortly after mutagenesis (aCGH was performed on the M3 generation in reference^1^) and has been assigned different alias names in previous work (including FN0175946 and VP02 in reference^2^). This mutant lineage was thereafter propagated from the M3 progeny and an advanced line of this mutant was used for the present study. The mutant was crossed to the accession ‘Noir 1’ [subline Noir 1-SGC-01^39^] which displays wild-type trichomes. F_2_ individuals were grown in the greenhouse and visually phenotyped for leaf trichome length. DNA was extracted from mutant and wild-type leaf tissue with a QIAGEN DNeasy kit and used to generate separate mutant and wild-type bulks. The two DNA bulks were genotyped with the SoySNP6K Beadchip (Illumina)^40^, and the data were used for BSA^41^ for the leaf trichome trait. To examine the root hairs, M92-220 and R59C46 were grown in the growth chamber at 28 °C in standard potting mix soil with 16 h of light and 8 h of darkness. At the first trifoliate growth stage the roots were sampled, washed, and images of the roots were taken with a 10x plan apo objective using a Nikon NiU Eclipse microscope equipped with a DS-Ri1 camera.

### Array comparative genomic hybridization

Array comparative genomic hybridization experiments were carried out on a microarray designed and manufactured by Agilent. Details about the array platform can be found at the National Center for Biotechnology Information Gene Expression Omnibus (GEO) (http://www.ncbi.nlm.nih.gov/geo) under accession number GPL22907. The aCGH methods for hybridizations, scanning, and data analysis were all performed as previously described^5^. The aCGH data from this study are available through the GEO accession number GSE118251.

### Determining deletion breakpoints

The exact edges of a genomic deletion cannot be determined by aCGH, and thus to determine the deletion breakpoints, it was necessary to generate and sequence amplicons spanning the deletion. Four pairs of primers were designed to span the deletion with the primer annealing sites positioned in a staggered manner just outside of the first and last aCGH probes located in the deletion. Two of the primer pair combinations successfully generated amplicons across the deletion break in the mutant line R59C46 while not generating amplicons in the wild-type M92-220 (see Supplementary Fig. S2). The amplified product was purified using a QIAquick PCR Purification Kit (QIAGEN) and then sequenced at the University of Minnesota Genomics Center (UMGC). The exact deletion break points were determined through an alignment of the junction amplicon sequence to the reference sequence Glyma.Wm82.a2.v1.1^28^.

### Testing co-segregation of the deletion in segregating F_2_ individuals

Co-segregation of the chromosome 6 deletion and the short trichome phenotype was tested on segregating F_2_ individuals. A co-dominant PCR assay was created for the co-segregation testing using the two primers (see Supplementary Table S3) flanking the interval and a third primer positioned just inside of the deletion interval unique to wild-type individuals (see Supplementary Fig. S2). The two forward primers, one for wild-type and one for mutant, shared the same reverse primer. The wild-type amplicon was longer than the mutant amplicon, and it was anticipated that if equal ratios of primers were used, the wild-type amplicon would not be preferentially amplified. Thus, in order to have roughly equal amplification of both bands, the ratio of the wild-type forward primer to the mutant forward primer was increased to be four to one. F_2_ individuals were grown in the greenhouse and were visually phenotyped for trichome length. Leaf tissue was collected from 96 F_2_ individuals and Noir1-SGC-01 and genomic DNA was extracted using a QIAGEN DNeasy kit. The co-dominant primer triple was used to screen the 96 F_2_ individuals as well as R59C46, ‘Noir 1’, and M92-220.

### Nuclear area measurements

Nuclear size data was obtained from fully expanded soybean leaves of R59C46 and M92-220 plants grown in a growth chamber at 28 °C in standard potting mix soil with 16 h of light and 8 h of darkness. Two fully expanded leaves from three individual plants were collected from each genotype. A 1 cm^2^ piece of leaf tissue from the center of the leaf was fixed, processed, and stained with 4′,6-diamidino-2-phenylindole (DAPI) according to the protocol by Downes *et al*.^25^. Ten DAPI stained trichome nuclei were measured from each 1 cm^2^ leaf fragment. All images were acquired with a 40x objective using a Nikon NiU Eclipse fluorescent microscope (Ex 325–375 nm, Em 435–485 nm) equipped with a DS-Qi1Mc monochrome camera and run by the NiS Elements AR software. Images were collected as a stack of 3–10 optical scans and compiled by the EDF module. Nuclei area was measured using the measurement tool of the Elements software. An ANOVA was performed, followed by Tukey’s HSD test using the statistical software R (version 3.5.3). Figures were created using the package “ggplot2” (version 3.1.0) of the statistical software R (version 3.5.3). Images were not adjusted when taking nuclear area measurements. However, for the visualizations shown in Fig. 2d,e, brightness, contrast and tonal range were adjusted using the levels tool in Adobe Photoshop.

### Stem trichome images

The stem trichome images were acquired using the Nikon SMZ18 stereo microscope equipped with the DS Ri2 colour camera and run by the NiS Elements AR software. The total image magnification was 15x. For scanning electron microscopy (SEM), leaf pieces were fixed for at least 1 h under vacuum while submerged in 2.5% glutaraldehyde buffered with 0.1 M sodium cacodylate at room temperature, then stored overnight at 4 °C. Samples were rinsed in buffer, postfixed in 1% osmium tetroxide in buffer overnight at 4 °C, rinsed in ultrapure water (NANOpure Infinity®; Barnstead/Thermo Fisher Scientific; Waltham, Maryland), and dehydrated in an ethanol series. Samples were then processed in a critical point dryer (Autosamdri-814; Tousimis; Rockville, Maryland). Material was mounted on aluminum stubs using double-sided adhesive carbon tabs and colloidal graphite, sputter-coated with gold-palladium, and observed in a scanning electron microscope (S3500N; Hitachi High Technologies America, Inc.; Schaumberg, Illinois) at an accelerating voltage of 10 kV.

### Measurement of leaf trichome length and diameter at the base for R59C46 and M92-220

Trichome length measurements for the R59C46 and M92-220 were taken from two fully expanded leaves from three individual plants for each line from three locations within a 1 cm^2^ leaf piece from the center region of the middle leaflets. Five trichomes per location were measured. All plants were grown in the growth chamber under a 16 hr photoperiod. Measurements were also taken of the trichome base diameter for R59C46 and M92-220. SEM images of trichomes were taken of the two lines, and ImageJ^42^ software was used to measure the base of the trichome tubular shaft in the region of the basal cells. For R59C46, 65 trichomes were measured, and for M92-220, 33 trichomes were measured. Trichomes from both the top and bottom side of the leaf were used. All plants were grown in the growth chamber under a 16 hr photoperiod. An ANOVA was performed, followed by Tukey’s HSD test using the statistical software R (version 3.5.3). Figures were created using the package “ggplot2” (version 3.1.0) of the statistical software R (version 3.5.3).

### Gene validation using CRISPR/Cas9 induced mutations

Additional mutated alleles were generated using CRISPR/Cas9 in order to validate the candidate gene. A CRISPR/Cas9 construct (see Supplementary Fig. S8) was created using the following methods. The soybean ubiquitin-3 promoter (GmUbi-3P) was amplified from pGmubi^43^. The reverse primer used in the reaction added a destination vector overhang for In-Fusion© cloning (Clontech), and the forward primer used in the reaction added an overhang for the selected terminator and both *Avr*II and *Nhe*I restriction sites. The terminator, RbcsT, was amplified from pGmute^44^. GmUbi-3P and RbcsT were assembled in a shuttle vector pSce^45^, separated by restriction sites *Avr*II and *Asc*I. Humanized *Staphylococcus pyogenes* (Spy) Cas9 was supplied by Addgene in plasmid #41815. SpyCas9 was amplified with the HiFi HotStart ReadyMix (Kapa Biosystems) using primers that overhang GmUbi3P and RbcsT at the *Avr*II and *Asc*I sites, respectively. A nuclear localization signal from Glyma.06g207800 was synthesized as a 63-nucleotide single-stranded oligonucleotide and added to Cas9 at the C-terminus. The shuttle was linearized with *Avr*II and *Asc*I, and the SpyCas9 amplicon was inserted using NEBuilder (New England BioLabs Inc). Then the cassette was removed with I-*Sce*I and moved into pStuH. The biolistic transformation vector pStuH is an adaptation of the pMECA^45^ vector system with the addition of *hph* for hygromycin B resistance, which is under the control of the *Solanum tuberosum* Ubiquitin 3 (Stubi-3) promoter and terminator^46^. MtU6 promoter was amplified with a forward primer that added homology to pStuHSpyCas9 at the *SpeI* restriction site (see Supplementary Table S4). Spy sgRNA scaffold^47^ was synthesized as a single-stranded oligonucleotide with an overhang at its 3′ end with homology to pStuHSpyCas9 at the *SpeI* cut site.

Geneious (Geneious R8-R11, Biomatters Ltd.) was used to select a target within the first exon of the candidate gene Glyma.06g145800. Potential targets for CRISPR editing were evaluated on three criteria: predicted on-target activity, target specificity, and likelihood to cause a functional disruption. The selected target sequence has a high specificity score of 95.24%^48^. Two predicted off-target sites are located in gene coding sequences, but both have mis-matches proximal to the Protospacer adjacent motif (PAM) site, making these potential sites very unlikely to be modified. The predicted activity of the target was 0.384, on a scale from 0 to 1^49^. The selected guide RNA (gRNA) for the candidate gene Glyma.06g145800 (see Supplementary Table S4) was synthesized with 18 bp overhangs for MtU6 and the scaffold, taking care to complement the other single-stranded oligonucleotide. pStuHSpyCas9 was linearized with *Spe*I, and the MtU6 gRNA and the scaffold were incorporated in one NEBuilder (New England BioLabs Inc.) reaction to create pStuHSpyC9xCPR5 (see Supplementary Fig. S3).

The soybean cultivar ‘Jack’^50^ was used for biolistic transformation. Soybean somatic embryos were transformed by particle bombardment as described by Trick *et al*.^51^ with modifications as described previously^52^. Briefly, 42 ng of pStuHSpyC9xCPR5 were precipitated on 0.6-µm diameter gold particles. Selection followed in liquid FNL medium^53^ with 20 µg mL^−1^ hygromycin-B.

At eight weeks post-bombardment, ten events were identified as hygromycin-resistant. Five of these transformation events were verified using Long-Range PCR with Promega GoTaq® Long PCR Master Mix and primers LT-PCR-1 and LT-PCR-2 (see Supplementary Table S4) to test for the presence of Cas9. Each of those five events was confirmed to express Cas9 by RT-PCR with primers RT-PCR-1 and RT-PCR-2 (see Supplementary Table S4). At ten weeks post-bombardment, the five verified events were analyzed using Tracking of Indels by DEcomposition (TIDE) analysis software^29^. Events were histodifferentiated in SHaM medium for five weeks to obtain mature somatic embryos, and then the embryos were desiccated for one week. Next, the embryos were germinated on MSO medium, and finally the plantlets were hardened off in soil boxes^54^. The T_0_ events were visually phenotyped after the V7 (seventh trifoliate) growth stage.

DNA was extracted from the T_1_ leaf tissue (Promega Wizard Genomic DNA Purification Kit), and PCR was used to generate amplicons spanning the CRISPR target site with primers cpr5_1180F and cpr5_2082R (see Supplementary Table S4). Amplicons were then Sanger sequenced at the UMGC, and the sequences were analyzed to determine the mutation status of the T_1_ individuals. DNA was also extracted from individual T_2_ plants derived from heterozygous T_1_ individuals, and the plants were genotyped using the same method used to genotype the T_1_ individuals. Trichome length measurements for three ‘Jack’, and a total of 30 T_2_ CRISPR/Cas9 mutants across the 10 genetic classes were taken on the adaxial side of the leaf. Trichomes were measured on three fully expanded leaves per individual. On each leaf, measurements were obtained from four locations within a 1 cm^2^ leaf piece from the center region of the middle leaflet. All length measurements were obtained using the Nikon NiS Elements AR software. All plants were grown in the growth chamber under a 16 hr photoperiod. As the alleles found in xCPR5 #3-4 and xCPR5 #3-5 families were identical, only individuals from xCPR5 #3-5 family were examined. An ANOVA was performed, followed by Tukey’s HSD test using the statistical software R (version 3.5.3). Figures were created using the package “ggplot2” (version 3.1.0) of the statistical software R (version 3.5.3).

## Supplementary information

Supplemental Files

## Data Availability

The aCGH microarray data is available through GEO accession number GSE118251. Information about this array platform can be found in accession number GPL22907. Both of these can be accessed through the GEO web-site (http://www.ncbi.nlm.nih.gov/geo).
