# Supplementary material for: Functional analysis and development of a CRISPR/Cas9 allelic series for a CPR5 ortholog necessary for proper growth of soybean trichomes
Source: Sci Rep. 2019 Oct 14;9:14757. doi: 10.1038/s41598-019-51240-7 (PMC6791840; doi:10.1038/s41598-019-51240-7)
Supplement: Supplementary file 1 — Supplemental Files [file 41598_2019_51240_MOESM1_ESM.pdf]

Supplementary Material

Functional analysis and development of a CRISPR/Cas9 allelic series for a CPR5 ortholog necessary for proper growth of soybean trichomes

*Benjamin W. Campbell[1], Jacob W. Hoyle[2], Bruna Bucciarelli[1,3], Adrian O. Stec[1], Deborah A. Samac[3,4], Wayne A. Parrott[2], *Robert M. Stupar[1]

[1] Department of Agronomy and Plant Genetics, University of Minnesota, St. Paul, MN, U.S.A.

[2] Department of Crop and Soil Sciences, University of Georgia, Athens, GA, U.S.A.

[3] USDA-ARS-Plant Science Research Unit, St. Paul, MN, U.S.A.

[4] Department of Plant Pathology, University of Minnesota, St. Paul, MN, U.S.A.

* Co-corresponding authors:

Benjamin W. Campbell campb964@umn.edu

Robert M. Stupar stup0004@umn.edu

Supplementary Table 1. List of deletions in R59C46 detected by array Comparative Genomic Hybridization (aCGH)

Chromosome	Start Probe	End Probe	Type	Notes
Gm03	22444873	22783786	Homozygous deletion in genic region	
Gm03	39355165	39364339	Homozygous deletion in genic region	
Gm06	4238463	4238908	Heterozygous deletion in non-genic region	
Gm06	11844851	11902734	Homozygous deletion in genic region	Causative deletion
Gm08	11460811	11508424	Homozygous deletion in genic region	
Gm12	10379033	10381278	Homozygous deletion in non-genic region	

Supplementary Table 2. Gene models located in the chromosome 6 deletion (Gm06: 11844488-11903108; Glyma.Wm82.a2.v1) in R59C46. The candidate gene is Glyma.06g145800.

Gene Name	Position (Genome version Glyma.Wm82.a2.v1)	Annotation	Best Arabidopsis TAIR10 hit	Best arabidopsis TAIR10 hit symbol
Glyma.06g145300	Gm06:11849535..11851671	PF00141 (Peroxidase)	AT5G05340.1 (Peroxidase superfamily protein)	
Glyma.06g145400	Gm06:11863821..11866022	PF03195 (Protein of unknown function DUF260)	AT2G40470.1 (LOB domain-containing protein 15)	ASL11, LBD15
Glyma.06g145500	Gm06:11873447..11878561	PF00560 (Leucine Rich Repeat), PF08263 (Leucine rich repeat N-terminal domain), PF00069 (Protein kinase domain)	AT5G10020.1 (Leucine-rich receptor-like protein kinase family protein)	
Glyma.06g145600	Gm06:11879451..11879931			
Glyma.06g145700	Gm06:11882177..11896120	PF03109 (ABC1 family)	AT5G64940.1 (ABC2 homolog 13)	ATATH13, ATH13, ATOSA1, OSA1
Glyma.06g145800	Gm06:11896583..11900105		AT5G64930.1 (CPR5 protein, putative)	CPR5, HYS1

Supplementary Table 3. PCR primers designed to amplify across the chromosome 6 deletion (Gm06: 11844488-11903108; Glyma.Wm82.a2.v1) in mutant R59C46.

Primer Name	Primer Sequence	Notes
"Primer 1" B271F	TTTTTCATGCCTAGACGTTGG	Use with B271R. Primer to span deletion on Chr 6 for the R59C46. Mutant 586 bp amplicon
"Primer 2" B271R	TTTCTGTTGAGTTTTTGTTAAACACC	Use with B271F. Primer to span deletion on Chr 6 for the R59C46. Mutant 586 bp amplicon
"Primer 3" B272 F2 WT	AAGAGCCACACAAATTGATGC	Primer to amplify wild-type band 955 bp (vs mutant 586 bp) when used with B271R (To make primer triple use: B271F, B272 F2 WT, B271R)

Supplementary Table 4. Sequences and PCR primers used in the creation and testing of the CRISPR/Cas9 construct

Primer Name	Primer Sequence	Notes
gRNA for GmCPR5	GGCGGCGAACAAGAACTCTA	guide RNA sequence
LT-PCR-1	CAGATCCGTTGACAAAAAGCCT	Used in Long-Range PCR to detect Cas9
LT-PCR-2	CCATTTCCATTTCACAGTTCG	Used in Long-Range PCR to detect Cas9
RT-PCR-1	CATATGATCAAATTTCGGGGACACTTC	Used in RT-PCR to test for Cas9 expression
RT-PCR-2	AAAGGTCTGCGTACTGGTCGCC	Used in RT-PCR to test for Cas9 expression
cpr5_1180F	CTTCACTGAAATTGCGACCC	Use to genotype the CRISPR target site
cpr5_2082R	TTGTGGCCAAAATCAGGG	Use to genotype the CRISPR target site
MtU6_Promoter_ Primer	TAACTATGTGCTTTGGATCTGCCCAAT GCCTATCTTATATGATCAATGAGG	Used to amplify the MtU6 promoter

a

b

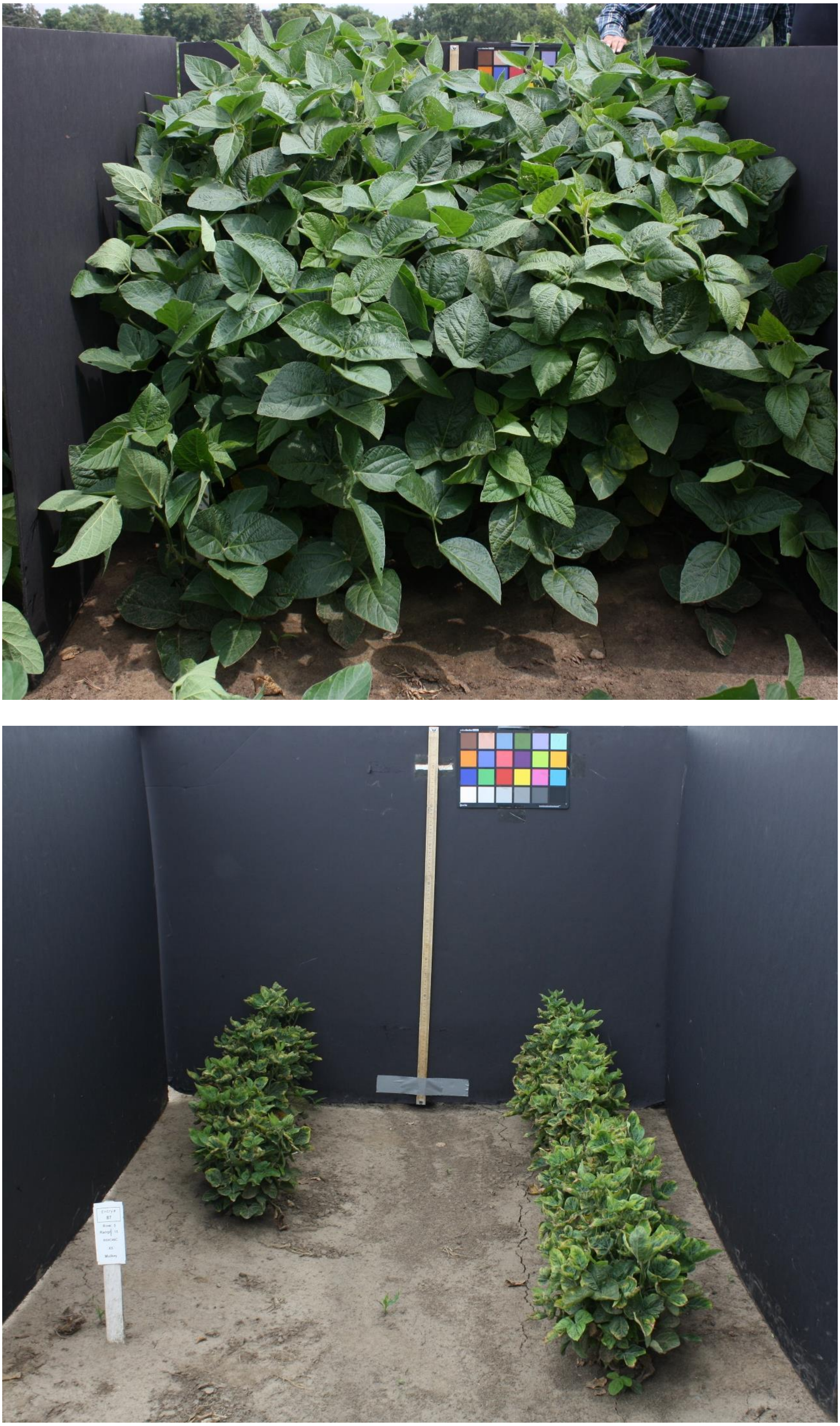

Supplementary Fig. S1. Relative growth and stature of wild-type M92-220 (a) and mutant R59C46 (b) when grown in field conditions. A meter stick is shown to scale the sizes of both images, though only the top of it is visible in part (a).

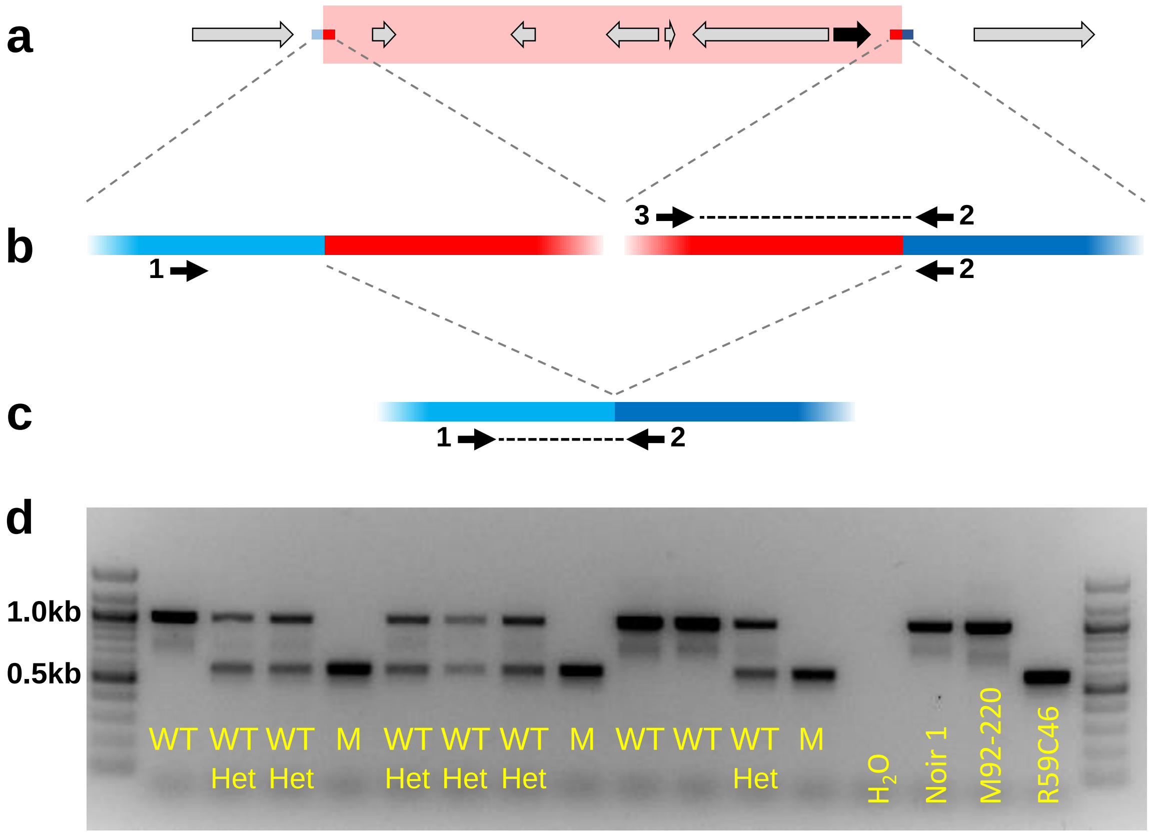

Supplementary Fig. S2. The fast neutron induced deletion detected on chromosome 6, the approximate positions of the primers used to span the deletion, and the co-segregation of the chromosome 6 deletion with the trichome phenotype. (a) The positions of two genes outside of the deletion and six genes within the deletion are indicated with arrows. The solid black arrow is used to indicate the candidate gene. The deletion is indicated by a light red box. (b) and (c) a magnification of the sequences at the edges of the deletion in wild-type and mutant plants, respectively. The sequences upstream and downstream of the deletion are colored in light blue and dark blue, respectively, and the deleted sequence is colored in red. The position and direction of the primers used for PCR are indicated with arrows. Primers 1 and 2 were used to amplify across the deletion. Primers 2 and 3 were used to generate an amplicon for the wild-type locus (without the deletion). The deletion was found to be a simple deletion with no additional sequence found in the deletion and no complex rearrangements. (d) PCR was performed on the segregating F_2 population to assay co-segregation of the deletion and the trichome phenotype. Primers 1, 2, and 3 were used together to generate a primer triple. The primers 1 and 2 generated a 586 bp amplicon across the chromosome 6 deletion. Primers 2 and 3 generated a 955 bp amplicon in wild-type individuals. Both amplicons were visible in heterozygous individuals. Mutant individuals are labeled as “M”, homozygous wild-type individuals are labeled as “WT”, and wild-type individuals that are heterozygous for the deletion are labeled as “WT Het”. Perfect recessive co-segregation of the deletion and the mutant phenotype was observed among the 96 F_2 individuals tested. From left: 12 progeny, no template control, ‘Noir 1’, M92-220, and R59C46.

```
Wm82 Gm06:11844458-11844487 CTCATTTAATTTGATGAGTTTTTATGTGAG
Wm82 Gm06:11903109-11903138                               CAATCGATATTATCTTAAATAGTAATTGAT
R59C46 at the Gm06 Deletion CTCATTTAATTTGATGAGTTTTTATGTGAGCAATCGATATTATCTTAAATAGTAATTGAT
```

Supplementary Fig. S3. Sequence at the junction of the two sides of the chromosome 6 deletion in R59C46. The light blue box indicates the sequence upstream of the deletion, and the dark blue box indicates the sequence downstream of the deletion. The figure shows 30 bp upstream and downstream from the deletion (Gm06: 11844488-11903108) which perfectly match with the corresponding sequences in Glyma.Wm82.a2.v1.1. No insertions, inversions, or translocations were observed within the deletion.

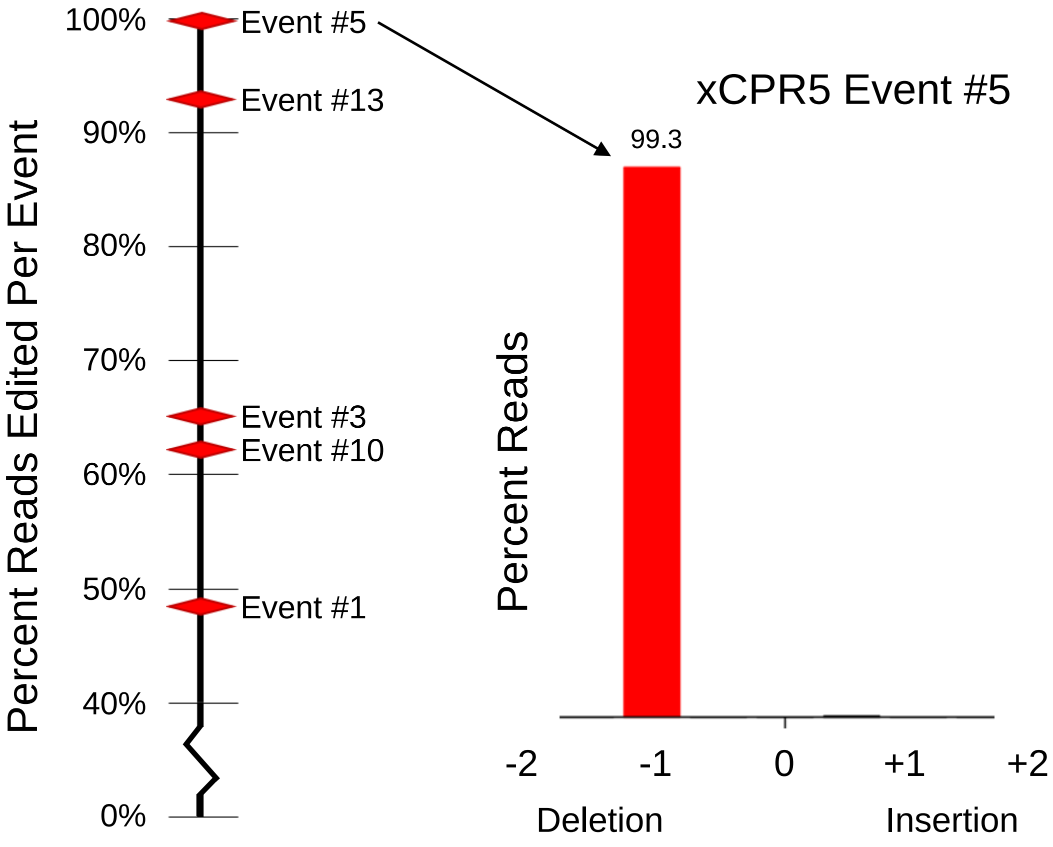

Supplementary Fig. S4. Total editing in five xCPR5 events at 10 weeks, as determined by TIDE. Red diamonds represent individual transgenic events. The panel on the right shows the relative frequency of indel sizes from transgenic event #5. Almost all of the indels in this event (99.3%) were 1 bp deletions.

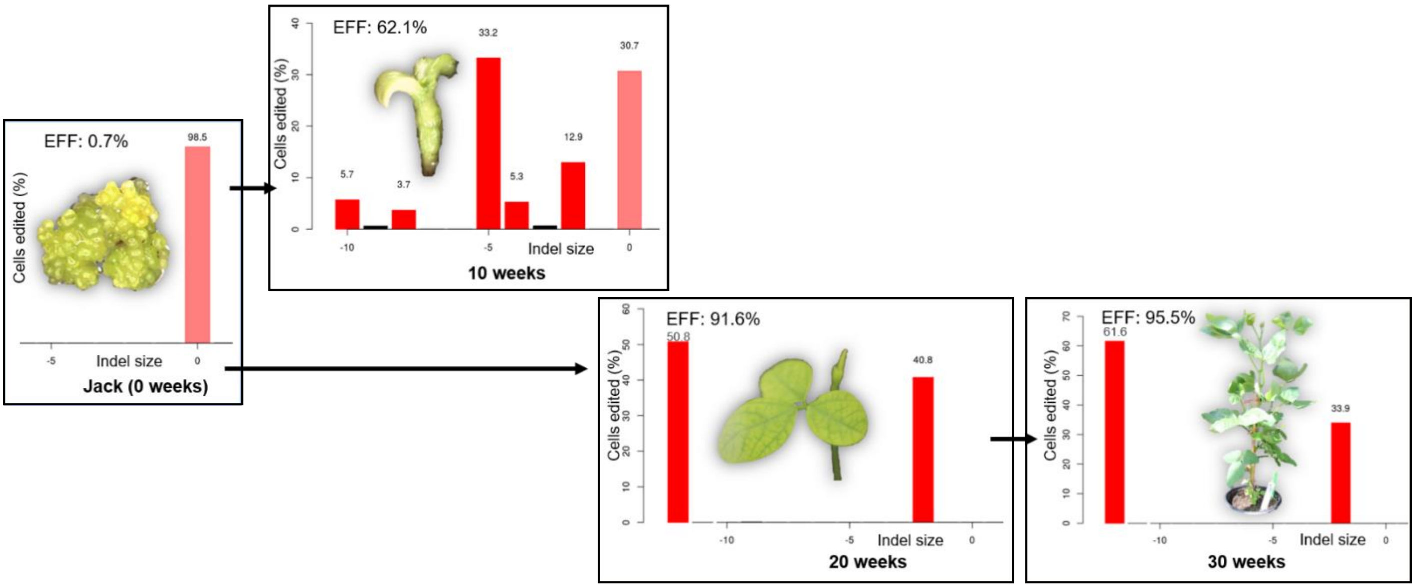

Supplementary Fig. S5. Editing over time in xCPR5 #3-1. TIDE output shows the composition of indels detected in the amplicons produced at the target site in *GmCPR5*. Photographs indicate developmental stage and phenotype of the event. The timeline is approximately: 0 weeks for the embryogenic cell line; 10 weeks for the mature somatic embryo; 20 and 30 weeks for plants.

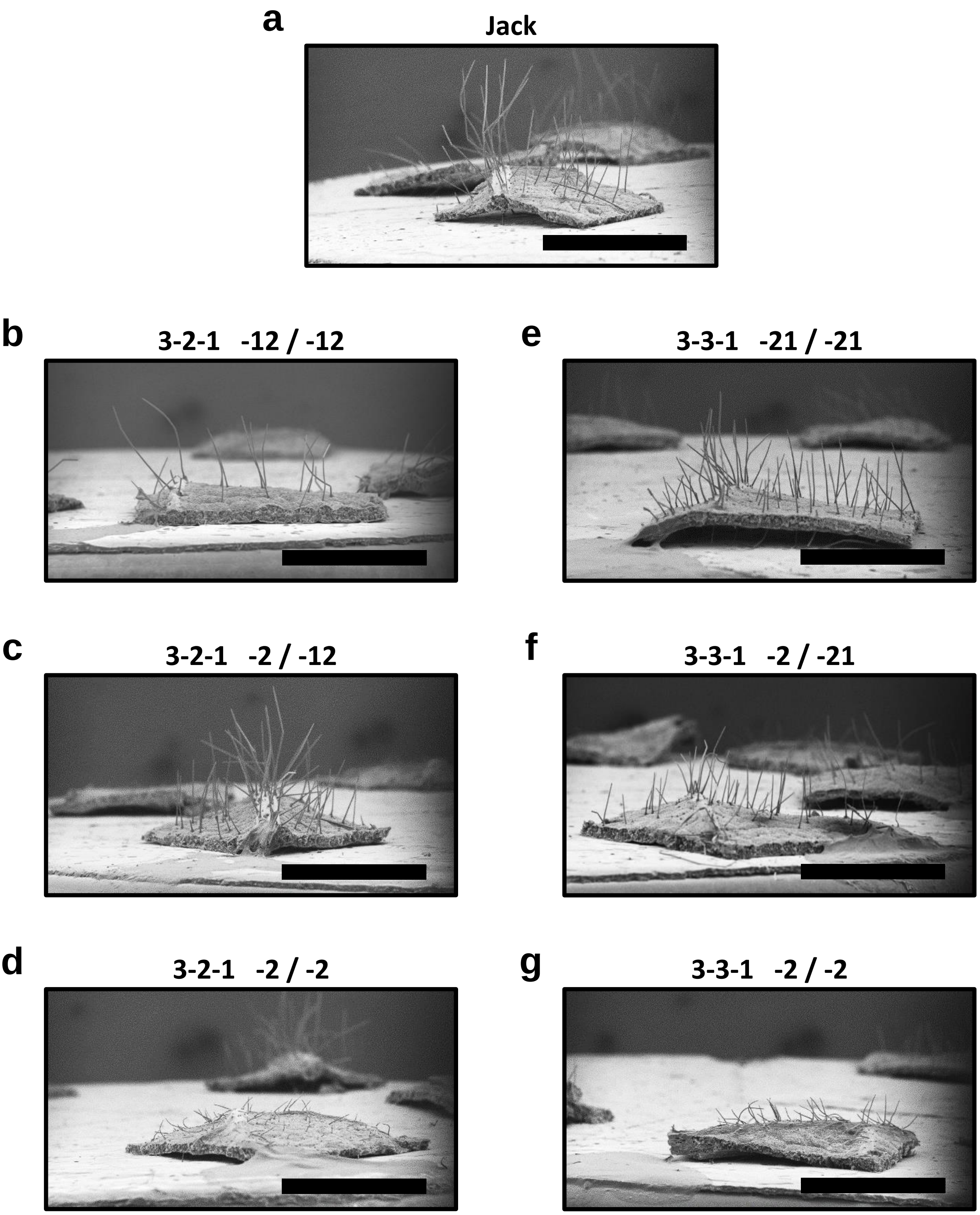

Supplementary Fig. S6. Scanning electron microscope trichome images of (a) ‘Jack’ and segregating T_2 mutant progeny with attached image labels indicating the source heterozygous T_1 individual and the genotype of the displayed T_2 progeny: (b) 3-2-1 with -12 / -12, (c) 3-2-1 with -2 / -12, (d) 3-2-1 with -2 / -2, (e) 3-3-1 with -21 / -21, (f) 3-3-1 with -2 / -21, and (g) 3-3-1 with -2 / -2. The size bar in each of the images is 2.5 mm.

Consensus	SSKSKQKGKRVSFKRRNPRVRFGPVRRHRGNNVDTIGLPLGMSFAAVMAQ
Glycine max Wild-Type	RKEN.G...AAA........L---.....A....................
xCPR5 #3-2	RKEN.G...AAA......**Sxx**---**xx**...A....................
xCPR5 #3-3	RKEN.G...AAA.......**xx**---**xxxxx**A....................
Cajanus cajan	.R.G.G...GAA.....S..L---.....A....................
Vigna radiata var. radiata	.R.R.S...KL.......S.V---.....A....................
Vigna angularis	.R.R.S...K........S.V---.....T....................
Phaseolus vulgaris	--.RRG.S...LL.....S.V---.....T...N....L..I.....T..
Medicago truncatula	--RT.V...GIAC.......S---...T.....AA..F.....V......
Arachis ipaensis	VR.T.R..--.A........AV--A..K...-..E...............
Arachis duranensis	VR.T.R..--.A.....S..AV--A..K...-..E...............
Lupinus angustifolius	-.S.L....---I.......HV--...IK.-D.SA...............
Gossypium barbadense	Y.S.V...M.L.S....L.........ADVLD..S.A..........V..
Durio zibethinus	..S.I.R.M.......T..........SEVRD..F.A..........V..
Herrania umbratica	C.S.I.R.TSLP....VS.........SKVGD.ES.A..........V..
Theobroma cacao	C.S.I.R.T..P....IT.........SEVGD.ES.A..........V..
Corchorus capsularis	C.S.I.R.T...SR..F.......A..VEVGD.ES.A..........V..
Helianthus annuus	VLH.RT..V...NN......FS.GL..RN.GEA.ALA......I..FVV.

Supplementary Fig. S7. Amino acid alignment of *Glycine max*, CRISPR mutants, and *CPR5* orthologs surrounding the residues affected by CRISPR mutations. The figure contains sequence alignments of wild-type Glycine max *CPR5*, the in-frame CRISPR mutant alleles from xCPR5 #3-2 and xCPR5 #3-3, and *CPR5* orthologs from several species. In the species sequence alignments, the dots indicate conserved amino acids and dashes indicate spaces in the sequence alignment. Mutations in xCPR5 #3-2 and xCPR5 #3-3 are indicated by bold text. The deleted amino acids in xCPR5 #3-2 and xCPR5 #3-3 are indicated by a bold ‘x’, and the amino acid sequence change of R to S is indicated in bold in the xCPR5 #3-2 sequence. The two vertical grey boxes highlight conserved amino acids that were deleted in xCPR5 #3-2 and #3-3.

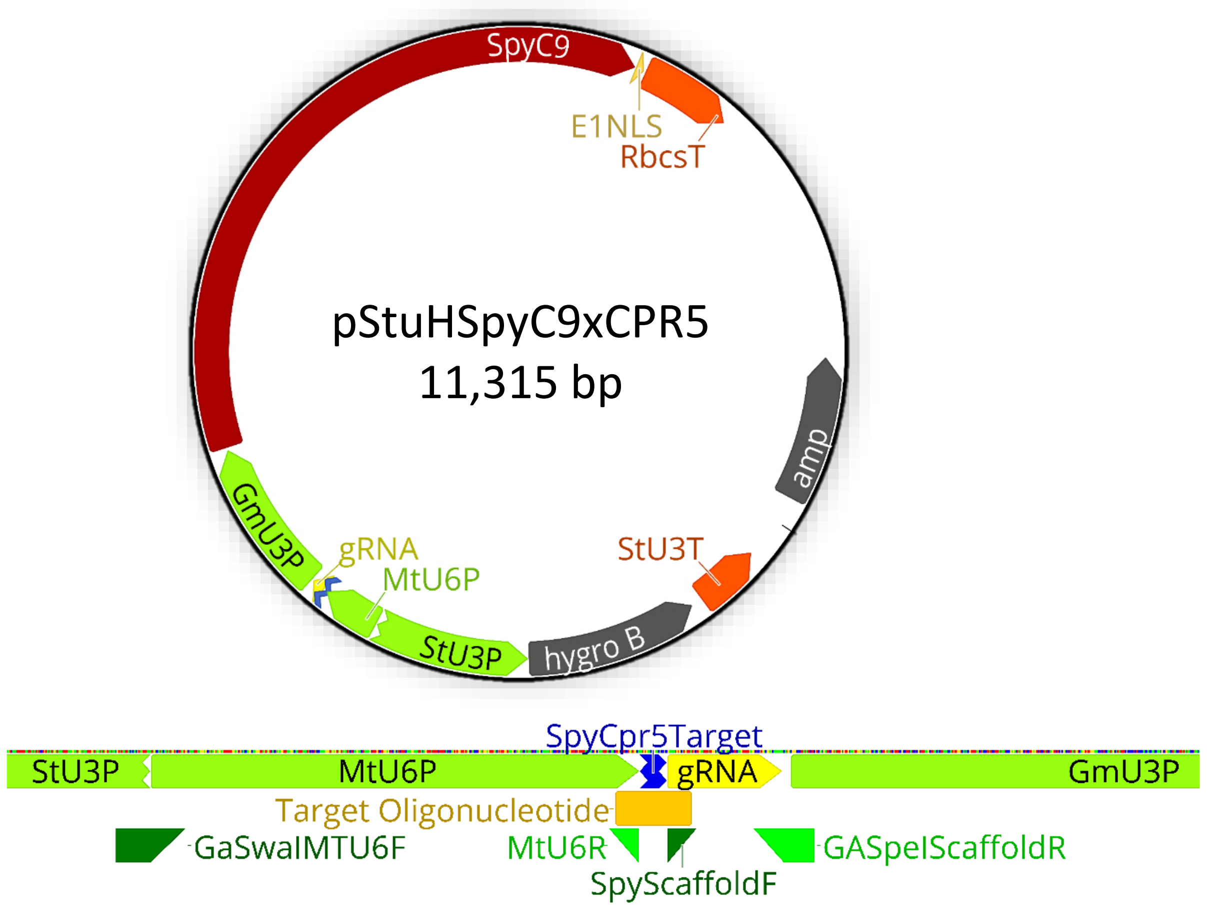

Supplementary Fig. S8. *GmCPR5* CRISPR knockout construct and details of the gRNA cassette.
